# Neurotensin Regulates Primate Ovulation Via Multiple Neurotensin Receptors

**DOI:** 10.1210/endocr/bqaf041

**Published:** 2025-03-04

**Authors:** Andrew C Pearson, Jessica S Miller, Hannah J Jensen, Ketan Shrestha, Thomas E Curry, Diane M Duffy

**Affiliations:** Department of Basic and Translational Sciences, Eastern Virginia Medical School, Old Dominion University, Norfolk, VA 23501, USA; Department of Basic and Translational Sciences, Eastern Virginia Medical School, Old Dominion University, Norfolk, VA 23501, USA; Department of Basic and Translational Sciences, Eastern Virginia Medical School, Old Dominion University, Norfolk, VA 23501, USA; Department of Obstetrics and Gynecology, University of Kentucky, Lexington, KY 40506, USA; Department of Obstetrics and Gynecology, University of Kentucky, Lexington, KY 40506, USA; Department of Basic and Translational Sciences, Eastern Virginia Medical School, Old Dominion University, Norfolk, VA 23501, USA

**Keywords:** ovary, monkey, endothelial cell, sortilin, luteinization, angiogenesis

## Abstract

Neurotensin (NTS), a small neuropeptide, was recently established as a key paracrine mediator of ovulation. *NTS* mRNA is highly expressed by granulosa cells in response to the luteinizing hormone surge, and multiple NTS receptors are expressed by cells of the ovulatory follicle. To identify the role of NTS receptors NTSR1 and SORT1 in ovulation in vivo, the dominant follicle of cynomolgus macaques (*Macaca fascicularis*) was injected with either vehicle control, the general NTS receptor antagonist SR142948, the NTSR1-selective antagonist SR48692, or the SORT1-selective antagonist AF38469. hCG was then administered to initiate ovulatory events. Ovulation was successful in all control-injected follicles. Rupture sites were smaller or absent after injection with NTS receptor antagonists. Histological analysis of follicles injected with SR142948, SR48692, or AF38469 revealed increased red blood cell extravasation and pooling in the follicle antrum when compared to controls. NTS receptor antagonist-injected follicles also showed dysregulated capillary formation and reduced luteinization of the granulosa cell layer. Prior in vitro studies showed that NTS significantly increased monkey ovarian microvascular endothelial cell (mOMEC) migration, while decreasing monolayer permeability. The NSTR1 antagonist SR48692 or siRNA knockdown of NTSR1 abrogated the ability of NTS to stimulate mOMEC migration and to decrease monolayer permeability. Similar experiments performed with the SORT1 antagonist AF38469 or siRNA knockdown of SORT1 also resulted in ablation of NTS-mediated changes in migration and permeability after SORT1 signaling was impaired. Together, these data implicate both NTSR1 and SORT1 to be critical mediators of NTS-stimulated ovulation, luteinization, and angiogenesis of the ovulatory follicle.

The luteinizing hormone (LH) surge, which occurs midway through the primate menstrual cycle, is the primary endocrine stimulus of ovulation. Numerous LH-mediated changes in the cells of the ovulatory follicle result in the release of the oocyte at ovulation and the development of the corpus luteum, a transient endocrine structure that is necessary for fertility (1). Rupture of the ovulatory follicle occurs after the LH-stimulated removal of both cells and extracellular matrix located between the follicle antrum and the exterior of the ovary (1). The LH surge also triggers the process of luteinization, in which the steroidogenic theca and granulosa cells of the follicle undergo a phenotypic differentiation to produce and secrete progesterone (2). Luteinization involves structural reorganization, including hypertrophy of the luteinizing granulosa cells and formation of a new capillary bed in the previously avascular granulosa cell layer of the ovulatory follicle (1). These exquisitely controlled events culminate in ovulation and, concomitantly, the formation of the corpus luteum.

The LH surge triggers follicular cells, including granulosa and theca cells, to produce many paracrine mediators of ovulation (1, 3). Neurotensin (NTS), a 13 amino acid peptide, has recently been identified as a key paracrine mediator of ovulatory changes (4, 5). Highly expressed by granulosa cells, follicular NTS increases dramatically in response to the LH surge in humans, monkeys, rats, and mice (4-6). Evidence supporting NTS's crucial role as an ovulatory mediator was demonstrated in macaque preovulatory follicles, which were injected with an NTS-neutralizing antibody followed by administration of an ovulatory gonadotropin stimulus (4). Neutralization of NTS decreased follicle rupture, impeded ovulatory angiogenesis, and increased vascular permeability, resulting in excessive accumulation of red blood cells within the follicle antrum (4, 7).

NTS action is mediated via 3 receptors (8). Neurotensin receptor 1 (NTSR1) and neurotensin receptor 2 (NTSR2) are members of the 7 transmembrane domain superfamily of receptors which can couple with G proteins (9). SORT1, also known as sortilin or neurotensin receptor 3 (NTSR3), has a single transmembrane domain and belongs to the Vps10p domain family of sorting proteins, which play a critical role in intracellular protein trafficking (10, 11). NTSR1 and SORT1 each bind NTS with high affinity, while NTSR2 binds NTS with lower affinity (12). NTSR1, NTSR2, and SORT1 are expressed by cells of ovarian follicles from a variety of mammalian species. The highest follicular expression levels of NTS receptors have been reported for NTSR1 and SORT1, with very limited expression of NTSR2 noted (4-6, 13, 14).

While an ovulatory role for NTS has been established, the specific receptor(s) which mediate the ovulatory actions of NTS remain unknown. Systemic administration of the NTSR1 antagonist SR46982 to mice experiencing ovarian stimulation resulted in a reduced number of oocytes recovered from oviducts (14). However, direct action of NTS at NTS receptors on ovarian cells to promote ovulation has not been confirmed. The purpose of the present study was to identify the roles of NTSR1 and SORT1 in mediating NTS-stimulated ovulation in macaques, primates with menstrual cycles very similar to those of women.

## Materials and Methods

### Monkeys

Whole ovaries and follicular fluid were collected from adult female cynomolgus macaques (*Macaca fascicularis*, aged 4-8 years) at Eastern Virginia Medical School. All animal protocols were performed in accordance with the National Institutes of Health's Guide for the Care and Use of Laboratory Animals (15) and with the approval of the Institutional Animal Care and Use Committee of Eastern Virginia Medical School. Animals were monitored daily for menstruation, and the first day of menstruation was designated as day 1 of the menstrual cycle. Blood samples were collected under ketamine chemical restraint (10 mg/kg body weight) via femoral or saphenous venipuncture. Serum samples were stored at −20 °C. Serum 17 β-estradiol (estradiol) and progesterone levels were measured using the ADVIA Centaur CP Immunoassay System (Siemens). Surgeries were performed aseptically under isoflurane anesthesia followed by postoperative analgesia (buprenorphine, with either ketoprofen or meloxicam as needed) (16).

### Controlled Ovulation With Follicle Injection

Neurotensin receptor antagonists or vehicle were delivered directly to the follicular fluid of a naturally selected ovulatory follicle as previously described (17). Serum concentrations of estradiol and progesterone were monitored daily beginning 5 to 7 days after menstruation. Once serum estradiol levels increased above 150 pg/mL, recombinant human follicle-stimulating hormone (FSH) (60 IU, Follistim, Organon) and recombinant human LH (60 IU, Serono) were administered once a day for 2 days to maintain the healthy growth of the ovulatory follicle. The gonadotropin releasing hormone (GnRH) antagonist Acyline (60 μg/kg per day; Eunice Kennedy Shriver National Institute of Child Health and Human Development) was also administered daily to prevent an endogenous LH surge. On the following day, surgery was performed to inject the ovulatory follicle or to perform ovariectomy to collect pre-LH/human chorionic gonadotropin (hCG) ovaries (n = 3). Control injections were performed with either sterile water (n = 2) or a nontargeting IgG (Abbiotec #254513, RRID:AB_2715565; n = 4) and are collectively referred to as Control. Additional follicles were injected with either the general NTS receptor antagonist SR142948 (Tocris Bioscience 2309, Bristol, UK; 100 µM, n = 4), the NTSR1 antagonist SR48692 (Tocris Bioscience 3721; 50 µM, n = 3), or the SORT1 antagonist AF38469 (Cayman Chemical 35530, Ann Arbor, MI; 0.25 µM, n = 3). Sterile water was used as the vehicle for the SR142948 and AF38469 injection solution. Sterile water with a final concentration of dimethyl sulfoxide <0.1% was used as the vehicle for the SR48692 injection solution. Injection of dimethyl sulfoxide to achieve a final follicle concentration of 0.1% (17, 18) or nontargeting IgG (RRID:AB_2715565) (4, 19, 20) have previously been demonstrated to have no adverse effect on ovulation. Directly after surgery, 1000 IU hCG was administered intramuscularly to initiate ovulation. Ovariectomy was performed 48 hours after hCG to remove the injected ovary, with ovulation expected at around 40 hours (21). Ovaries were fixed with 10% formalin, embedded in paraffin, and sectioned in their entirety at 5 µm. Information regarding IgG-injected controls (4, 19, 20), as well as limited histological analysis of water-injected follicles and SR142948-injected follicles (7), has been previously reported.

### Histology

Whole ovaries were fixed in 10% formalin for 24 hours and embedded in paraffin, oriented such that sections included the follicle apex and follicle wall opposite the apex at the maximal follicle diameter (4). Ovaries were serially sectioned at 5 μm, with each section retained in order. Every fifth section was deparaffinized in xylene, rehydrated through a graded ethanol series, stained with hematoxylin and eosin (Sigma-Aldrich), dehydrated through a graded ethanol series, and permanently coverslipped (Permount #SP15, Thermo Fisher Scientific). Ovaries were evaluated by 2 independent observers.

Rupture site size was quantified by measuring the width on the section with the largest rupture site and counting the number of 5-μm sections where the rupture site was present to calculate the area of an oval. Unruptured follicles or follicles that ruptured to the interior of the ovary (termed aberrant rupture) were assigned a rupture area of 0 mm^2^.

Immunohistochemistry was performed and analyzed for detection of Von Willebrand factor (VWF) as a marker for endothelial cells as previously described (19) using an ovarian section containing both (1) the maximal diameter of the follicle and (2) rupture site or thinnest apical portion of an unruptured follicle. Briefly, sections of paraffin-embedded ovaries were heated, deparaffinized, rehydrated, blocked in phosphate-buffered saline containing 0.1% Triton X-100, incubated overnight with a rabbit polyclonal antibody against VWF (5 µg/mL; Agilent #A0082, RRID:AB_2315602), and color developed using a rabbit Vectastain ABC kit (Vector Laboratories). Slides were counterstained with hematoxylin, dehydrated, and permanently coverslipped.

Immunofluorescent staining for the detection of Ki67 and prostaglandin-endoperoxide synthase 2 (PTGS2, also known as COX2) was performed. Paraffin-embedded ovary sections were heated, deparaffinized, and rehydrated. For Ki67 staining, slides were exposed to basic antigen retrieval (heating to 85-90 °C for 30 minutes in 10 mM Tris, 1.3 mM EDTA, 0.05% Tween 20). Sections were blocked first with Image-iT FX signal enhancer (Thermo Fisher Scientific, Waltham, MA) for 30 minutes at room temperature. Sections were then blocked for 30 minutes at room temperature in 5% normal goat serum in PBS. A Streptavidin/Biotin Blocking Kit (Vector Laboratories, Burlingame, CA) was used per the manufacturer's instructions to block endogenous biotin and streptavidin binding. Slides were incubated overnight at 4 °C with either Ki67 mouse monoclonal (Agilent, Santa Clara, CA; Cat# M7240, RRID:AB_2142367; 1:100, protein concentration of antibody solution not provided by supplier) or PTGS2 mouse monoclonal (Cayman Chemical, Ann Arbor, MI; Cat# 160112-1, RRID:AB_327868, 2 µg/mL) primary antibodies in goat serum blocking solution. Slides were then incubated in goat antimouse IgG antibody (H + L), biotinylated (Vector Laboratories, Cat# BA-9200, RRID:AB_2336171, 7.5 µg/mL) in goat serum blocking solution for 2 hours at room temperature. Biotinylated antibodies were then fluorescently tagged with AF488-conjugated streptavidin (Molecular Probes S11223, 4 µg/mL) for 30 minutes. Autofluorescence was quenched with double filtered 1% Sudan black in 70% methanol and slides were mounted with Prolong Gold antifade reagent with DAPI (Thermo Fisher Scientific, Waltham, MA). Omission of primary antibody served as a negative control. Slides were photographed using a Zeiss Observer Z1 microscope fitted with an AxioCam MRm camera and Zen software v3.8 for image acquisition (Zeiss, Oberkochen, Germany).

Granulosa cell layer thickness and endothelial cell invasion were assessed as quantitative metrics of luteinization as previously described (19). Directly across from the follicle apex (maximal rupture or thinnest apical tissue in unruptured follicles), the granulosa cell layer was measured from basal lamina to antral edge, and the distance from the basal lamina to the antral-most VWF-positive cell was also measured. Eight (8) replicate pairs of measurements were made per ovary. For each ovary, average measurements for granulosa cell layer thickness and endothelial cell invasion were determined, and the ratio of endothelial cell invasion/granulosa cell layer thickness was calculated.

Terminal deoxynucleotidyl transferase dUTP nick-end labeling (TUNEL) of ovarian tissues was performed using the DeadEnd Fluorometric TUNEL system according to kit instructions (#G3250, Promega, Madison WI). Stained tissues were mounted with diamidino-2-phenylindole (DAPI) (Prolong gold antifade mountant, #P36931; ThermoFisher Scientific) and photographed using a Zeiss Observer Z1 microscope fitted with an AxioCam MRm camera and Zen software v3.8 for image acquisition (Zeiss).

Unless otherwise stated, histology and immunohistochemistry were imaged by using an Olympus microscope with a DP70 digital camera system and associated software (Olympus, Melville, NY). Whole follicle images were assembled from multiple microscopic images of a single tissue section using Image Composite Editor (Microsoft Corp., Redmond, WA).

### Monkey Ovarian Microvascular Endothelial Cells

Ovarian stimulation was performed as previously described (16). Briefly, to stimulate the growth of multiple follicles, monkeys received FSH (Organon) and LH (Serono) for a total of 7 to 10 days. GnRH antagonist (Ganirelix; Organon) was also administered daily to prevent endogenous release of an ovulatory surge of LH. Serum estradiol and ovarian ultrasonography were assessed to ensure adequate follicular development. Thirty-six hours prior to aspiration, 1000 IU hCG (Ovidrel, EMD Serono) was administered via intramuscular injection. At surgery, follicle aspirates were collected from all ovarian follicles greater than 4 mm.

Ovarian microvascular endothelial cells were isolated from follicle aspirates to create replicating populations of primary monkey ovarian microvascular endothelial cells (mOMECs) as previously described (18). Aspirated cell suspension was plated on fibronectin-coated tissue culture treated flasks in microvascular endothelial cell optimized medium (EGM-2MV, Lonza) and grown to confluence. To enrich for endothelial cells, the cells were subjected to 2 rounds of CD31 Dynabead (Invitrogen) magnetic bead isolation following the manufacturer's protocol (18). The resulting cell population was >95% endothelial cells (18). Cells were grown in EGM-2MV medium (Lonza). Medium was changed to basal medium (EBM-2, Lonza) with 1% fetal bovine serum (Gibco) overnight prior to beginning experiments. Treatments were performed in basal medium. Replicate experiments were performed using mOMECs from different animals. All experiments were performed using cells passage 3 to 9.

### In Vitro Migration Assay

Migration of mOMECs in response to various stimuli was assessed as previously described (4). mOMECs were seeded directly onto cell culture inserts containing membranes with 8 µm pores (BD Biosciences, San Jose, CA), and recombinant human NTS (Bachem, Torrance, CA; 5 µM) with or without SR142948 (Tocris; 25 µM), SR48692 (Tocris; 10 µM), or AF38469 (Cayman; 0.1 µM) was added to basal medium in the well of the culture plate. After 24 hours, membranes were stained with hematoxylin and eosin (Sigma-Aldrich, St. Louis, MO). Images of migrated cells (5 images per membrane) were counted and averaged.

### In Vitro Transwell Permeability Assay

The ability of the horseradish peroxidase conjugated streptavidin (SHRP) to pass through an endothelial cell monolayer was tested in a transwell permeability assay (22). mOMECs were grown to ∼90% confluence on fibronectin-coated polycarbonate membrane cell culture inserts containing 0.4-µm pores (Corning, Corning, NY). A 24-well plate, which has the same surface area per well as the inserts, was also seeded with equal density of mOMECs in tandem to confirm the confluence of cells seeded on the opaque inserts. Once the desired confluence was reached, growth media were changed to basal media +1% fetal bovine serum overnight. Media were then changed to basal media alone or basal media containing NTS (Bachem #4008910, Torrance, CA; 5 µM) for 1 hour. Treatment media were removed, and inserts were transferred to a new 12-well plate containing fresh basal media. Media containing SHRP were added to the top of the inserts and incubated at 37 °C for 20 minutes. Inserts were carefully removed, and the media beneath the inserts were transferred to a 96-well plate in triplicate. 3,3′,5,5′-tetramethylbenzidine substrate (#860336; Sigma-Aldrich) was added to each well for 7 minutes at room temperature. Stop solution (2 N H_2_SO_4_ in water) was added to each well, and absorption at 450 nm (reference wavelength = 0) was measured on a plate reader (Tecan Sunrise, Männedorf, Switzerland). Absorption values of media from sample wells were compared to a standard curve of SHRP in basal media to determine SHRP concentration. Thrombin (1 U/mL) and dibutyryl-cAMP (db-cAMP, 10 µM) served as positive and negative controls, respectively (23, 24).

### Western Blot

Endothelial cell lysate preparation and western blotting were performed essentially as previously described (4). Protein from lysates of mOMECs were loaded onto a 4% to 12% polyacrylamide gradient gel (Invitrogen). Proteins were then transferred to a polyvinylidene fluoride membrane (Immobilon; Millipore, Billerica, MA). Membranes were blocked in 5% nonfat dry milk in Tris-buffered saline (#SC-24951, Santa Cruz Biotechnology) with 0.1% Tween 20. Membranes were probed with antibodies against NTSR1 (Thermo Fisher Scientific #PA3-214, RRID:AB_10979876, 1:500, protein concentration of antibody solution not provided by supplier), SORT1 (Sigma-Aldrich #HPA006889, RRID:AB_1080056, 0.4 µg/mL), or pan-actin (Millipore #MAB1501, RRID:AB_2223041, 5 µg/mL), then incubated with an antirabbit HRP-conjugated secondary antibody (Vector Labs #PI-1000, RRID:AB_2336198, 0.1 µg/mL). Protein bands were visualized with Amersham ECL Western Blotting Detection Reagents (Cytiva, Marlborough, MA).

### RNA Isolation, Amplification, and Quantitative Polymerase Chain Reaction

mOMECs were grown to ∼90% confluence prior to RNA isolation following the manufacturer's instructions using the RNeasy Mini Kit (Qiagen, Hilden, Germany). Sample RNA quality and quantity was measured using NanoDrop 1000 (NanoDrop Technologies, Wilmington, DE), and normalized quantities of mRNA were converted to cDNA per manufacturer's instructions using the Qiagen RT2 First Strand Kit and a Bio-Rad C1000 Touch thermal cycler (Bio-Rad Laboratories, Hercules, CA). Custom primers were ordered from Thermo Fisher Scientific (Table 1). Quantitative polymerase chain reaction (qPCR) was performed using CFX96 Real-Time System (Bio-Rad Laboratories) and FastStart SYBR Green Master (Roche Diagnostics GmbH; Mannheim Germany). The cycling conditions were as follows: 10 minutes at 95 °C; then 45 cycles of 15 seconds at 95 °C, 30 seconds at 55 °C ( β-actin (*ACTB*) and *NTSR1*) or 60 °C (*SORT1*), and 45 seconds at 72 °C. A melt curve was performed ranging from 65 °C to 95 °C. For confirmation of siRNA knockdown, the 2^−ΔΔCT^ method was used to compare *NTSR1* and *SORT1* cycle thresholds to the housekeeping gene, *ACTB*. Percent knockdown was then calculated by multiplying the fold change by 100 to obtain % remaining gene expression (25).

**Table 1.** Polymerase chain reaction primers

Target	Primer sequence (5′ to 3′)	Accession #
*NTSR1*	F-CGCCTCATGTTCTGCTACAT R-TTGATGGTGGAGCTGACGTA	XM_005569538.2
*SORT1*	F-GGGGGACGTTTCCTTTTTGC R-TCCGCCTGTGGTAGTGTAGA	XM_005542475.2
*ACTB*	F-ATCCGCAAAGACCTGT R-GTCCGCTAGAAGCAT	NM_001285025.1

### NTSR1 and SORT1 Silencing by Small Interfering RNA

mOMECs were seeded in Opti-MEM reduced serum media (Gibco) and transfected immediately using Lipofectamine RNAiMAX transfection reagent (Invitrogen) according to the manufacturer's protocol. Cells were transfected with 20 nM of a custom siRNA construct targeting *M. fascicularis SORT1* (Table 2, Thermo Fisher Scientific) with a nontargeting siRNA (siNC, Thermo Fisher Scientific). Additional cells were transfected with 50 nM of a custom siRNA construct targeting *M. fascicularis NTSR1* (Table 2, Genecust, Boynes, France). Cells were transfected for 24 hours before media was changed to EGM-2MV growth media (Lonza) until confluent. Cells were then trypsinized and seeded into the trans-well permeability assay or migration assay as described above. At the end of the assay, mRNA from the cells was assayed by qPCR to evaluate knockdown efficacy as described in RNA isolation, amplification, and quantitative PCR.

**Table 2.** siRNA constructs

Gene name	Sequence—sense (5′-3′)	Sequence—antisense (5′-3′)
*SORT1*	UUGUUGUUAGGUGUUCUUC[dT][dC]	GAAGAACACCUAACAACAA[dA][dT]
*NTSR1*	CUCAUGUUCUGCUACAUUU[dC][dG]	AAAUGUAGCAGAACAUGAG[dG][dC]

### Statistics

**Figure 1.**
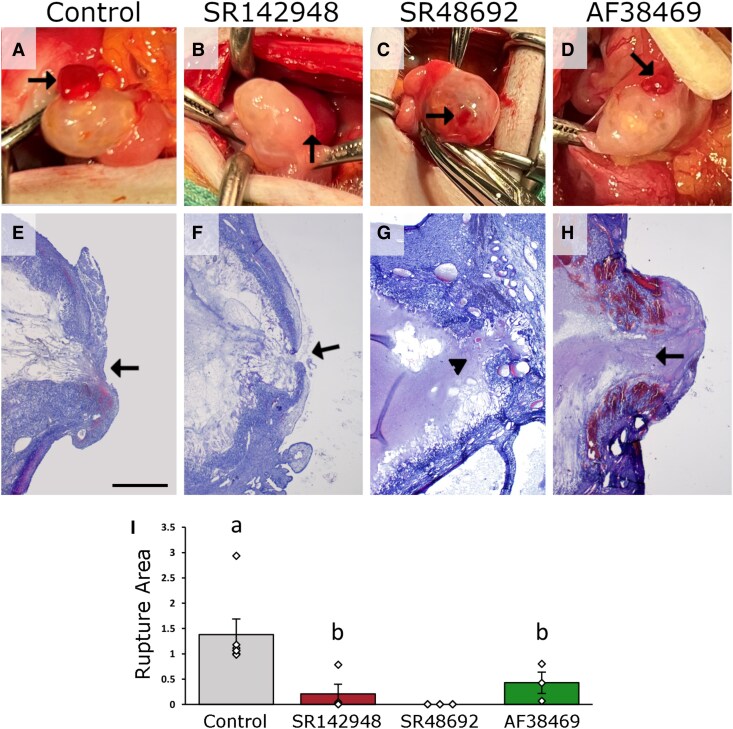
NTS receptor antagonists compromise follicle rupture. Monkey preovulatory follicles were injected with vehicle (Control; A, E), the general NTS receptor SR142948 (B, F), the NTSR1 antagonist SR48692 (C, G), or the SORT1 antagonist AF38469 (D, H); hCG was administered immediately thereafter, and ovariectomy was performed 48 hours later. (A-D) Ovaries just prior to ovariectomy and are at similar magnification. (E-H) Histological evidence of rupture in Control (E), SR142948 (F), and AF38469 (H) injected follicles (arrows); aberrant rupture into ovarian stroma after injection with SR48692 (G, arrowhead) is also shown. Images in E-H are at the same magnification, with bar in E = 1 mm. (I) Rupture site area (mm^2^) was quantified for follicles injected with vehicle (Control; n = 6) SR142948 (n = 4), SR48692 (n = 3), or AF38469 (n = 3). Diamonds indicate individual data points; absence of rupture or aberrant rupture was assigned a rupture area of 0 mm^2^. Data were assessed by ANOVA; groups with no common letter are different by the Duncan post hoc test, *P* = .0073. SR48692 group was excluded from statistical analysis because all ovaries had a rupture site area of 0 mm^2^.

Data were assessed for heterogeneity of variance by Bartlett tests. The Bartlett test for data in Fig. 1I yielded *P* < .05, so these data were log10 transformed before further analysis. To test differences between treatments, analysis was performed using 2-tailed paired Student t-tests or 1-way analysis of variance (ANOVA) with repeated measures, followed by the Duncan post hoc test as indicated in each figure legend. *P* < .05 was considered statistically significant, and significant *P* values are given in each figure legend. Statistical analysis was performed using StatPak version 4.12 software (Northwest Analytical, Portland, OR). Data are presented as mean ± SEM.

## Results

### NTS Regulates Follicle Rupture Via Multiple NTS Receptors

To determine the role of endogenous NTS in the regulation of ovulatory events, a large preovulatory macaque follicle was injected with vehicle (control), the general NTS receptor antagonist SR142948, the NTSR1 selective antagonist SR48692, or the SORT1 antagonist AF38469. hCG was then administered systemically to initiate NTS expression and other ovulatory changes. The ovary was removed 48 hours after follicle injection and hCG administration, with ovulation expected at about 40 hours (21).

Control follicles showed evidence of ovulation at ovariectomy, with an ovulatory stigmata protruding from the ovarian surface (Fig 1A). Histological assessment of control follicles confirmed the presence of a large rupture site connecting the follicle antrum to the space outside the ovary, with luteinizing tissue extending through the rupture site to ovarian surface (Fig 1E).

Follicles injected with SR142948 showed minimal disruption of the ovarian surface at ovariectomy (Fig 1B and 1I). Histological assessment of these follicles showed absent (n = 2) or small (n = 2; Fig 1F and 1I) rupture sites, with no luteinizing tissue protruding through the rupture site to the exterior of the ovary.

Follicles injected with SR48692 showed minimal or no disruption of the ovarian surface at the time of ovariectomy (Fig 1C and 1I). Histological assessment of these follicles demonstrated a lack of complete rupture to the exterior of the ovary. However, a breach in the follicle wall into the ovarian stroma surrounding the follicle was noted in SR48692-injected follicles (Fig 1G) and was termed aberrant rupture. Aberrant rupture into the ovarian stroma was noted in all SR48692-injected follicles, with 2 distinct aberrant rupture sites identified in a single SR48692-injected follicle.

Follicles injected with AF38469 showed evidence of follicle rupture at ovariectomy (Fig 1D). Histological assessment of these follicles confirmed the presence of a rupture site connecting the follicle antrum to the exterior of the ovary in all cases, with a small amount of luteinizing tissue extending to the exterior of the ovary (Fig 1H). Rupture sites in all AF38469-injected follicles were very bloody, with large vessels present in the stroma and notable red blood cell accumulation near the stigmata (Fig 1H).

NTS receptor antagonists decreased the overall frequency of follicle rupture after follicle injection and 48-hour exposure to hCG. Rupture, defined as connecting the follicle antrum to the exterior of the ovary, was noted in 100% of control-injected and AF38469-injected follicles (Table 3). In contrast, rupture occurred in only 50% of follicles injected with SR142948 (Table 3). Rupture connecting the follicle antrum to the ovarian exterior was absent in all follicles injected with SR48692 (Table 3).

**Table 3.** NTS antagonists disrupt follicle rupture in vivo

	Exterior rupture	Aberrant rupture	No rupture
Control	100%	0%	0%
SR142948	50%	0%	50%
SR48692	0%	100%	0%
AF38469	100%	0%	0%

Blockade of NTS receptors also decreased the size of rupture sites. Compared to control follicles, injection of SR142948 or AF38469 decreased the rupture site area (Fig 1I). At 48 hours after hCG, rupture connecting the follicle antrum to the ovarian exterior was absent in all follicles injected with SR48692, so all SR48692-injected follicles had an effective rupture size of 0 mm^2^ and were excluded from statistical analysis (Fig 1I).

### NTS Impacts Luteinization and Angiogenesis Via Multiple NTS Receptors

**Figure 2.**
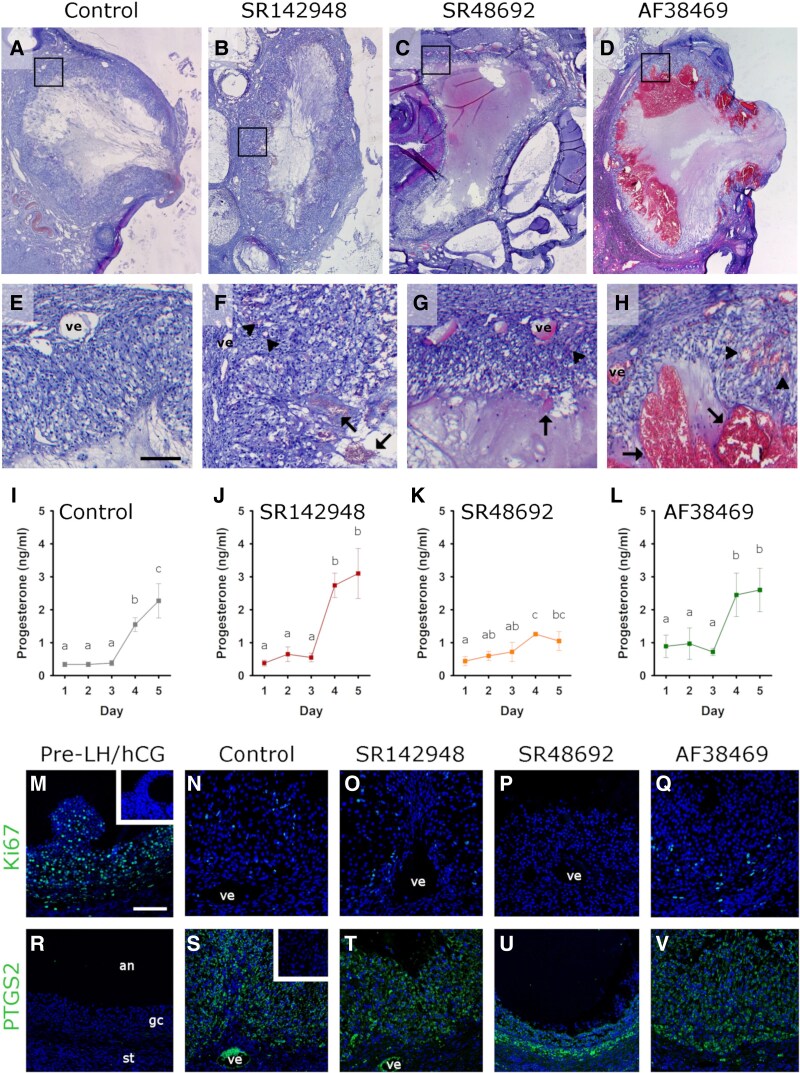
NTS receptor antagonists compromise follicle luteinization in vivo. The ovulatory follicle and surrounding ovarian tissue after follicle injection with vehicle (Control, A, E), the general NTS receptor SR142948 (B, F), the NTSR1 antagonist SR48692 (C, G), or the SORT1 antagonist AF38469 (D, H). All images A-D are at similar magnification. Black box in A-D shows the location of higher magnification images (E-H). All images E-H are the same magnification, with bar in image E = 100 µm. Stromal vessels (ve), red blood cell pooling in the follicle antrum (arrows), and red blood cell accumulation within the luteinizing granulosa cell layer (arrowheads) are indicated in follicles injected with SR142948 (F), SR48692 (G), and AF38469 (H). (I-L) Serum progesterone (ng/mL) during FSH and LH administration (Days 1-2), follicle injection and hCG administration (Day 3), and ovariectomy (Day 5) for monkeys receiving follicle injection of vehicle (Control, I; n = 6), SR142948 (J; n = 4), SR48692 (K; n = 3), or AF38469 (L; n = 3). On each day, no differences were detected between treatment groups by ANOVA (*P* > .05). Within each treatment group, ANOVA with 1 repeated measure detected differences between days (Control, *P* = .0000; SR142948, *P* = .0002; SR48692, *P* = .0389; AF38469, *P* = .0014). Within each treatment group, days with no common letters are different by Duncan's post hoc test, *P* < .05. (M-V) Ki67 immunostaining in a pre-LH/hCG follicle (M), Control follicle (N), and follicles injected with either SR142948 (O), SR48692 (*P*), or AF38469 (Q). PTGS2 immunostaining in a pre-LH/hCG follicle (R), Control follicle (S), and follicles injected with either SR142948 (T), SR48692 (U), or AF38469 (V). (M-V) use the same orientation as image in R, with antrum (an) at top, granulosa cells (gc) central, and follicular stroma (st) in the lower portion of the image. Vessels with luminal diameter >∼75 µm are marked (ve). PTGS2 antibody resulted in nonspecific staining of red blood cells (bright green), which is evident in G and H. Insets in M and S are staining controls from the same tissue without primary antibody. DAPI nuclear counterstain (dim blue). Images in M-V are representative of n = 3-4 follicles per treatment group and are shown at the same magnification; bar in M = 100 µm.

**Figure 3.**
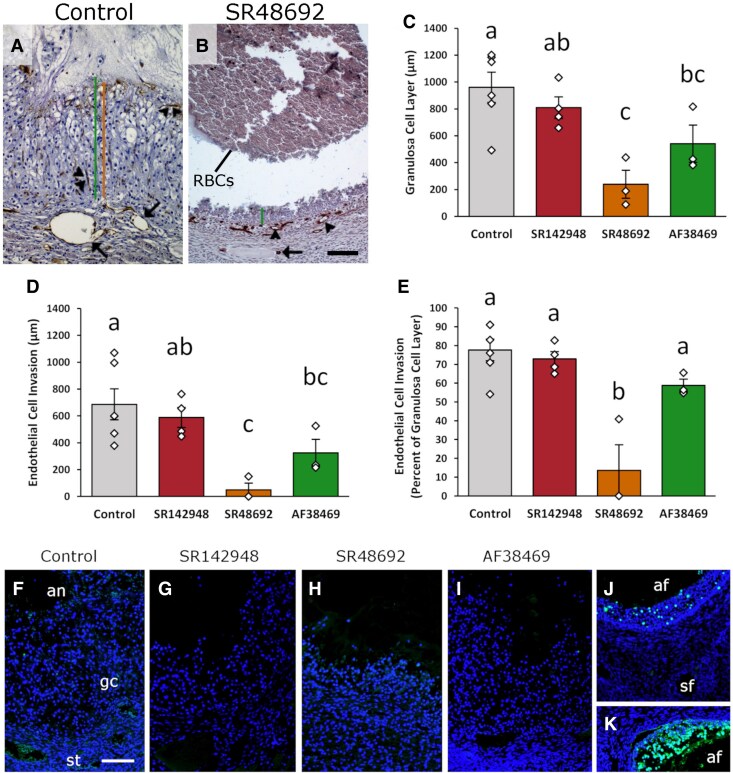
NTS receptor antagonists compromise luteinization and angiogenesis in vivo. (A, B) Examples measurements of granulosa cell layer thickness (green line, A and B) and endothelial cell invasion beyond the granulosa cell basal lamina (orange line, A only) in a control follicle (A) and SR48692-injected follicle (B). VWF immunodetection is brown, and hematoxylin counterstain is blue. Arrows indicate stromal vessels, single arrowheads indicate stromal endothelial cells, and double arrowheads indicate endothelial cells in the granulosa cell layer. Antral red blood cells (RBCs) are indicated in B. Magnification is the same in A and B; bar in B = 100 µm. Granulosa cell layer thickness (C), endothelial cell invasion (D), and the ratio of endothelial cell invasion as a percentage of granulosa cell layer thickness (E) for Control follicles and follicles injected with either SR142948, SR48692, or AF38469. Diamonds represent individual data points. Within C-E, groups with no common letters are different by ANOVA with 1 repeated measure (C, *P* = .0047; D, *P* = .0060; E, *P* = .0002) and Duncan's post hoc test, *P* < .05. Data are presented as mean ± SEM; n = 3-6 follicles per treatment group. (F-K) TUNEL staining for DNA fragmentation (bright green) in Control follicle (F) and follicles injected with either SR142948 (G), SR48692 (H), or AF38469 (I). (F-I) use the same orientation as image in F, with antrum (an) at top, granulosa cells (gc) central, and follicular stroma (st) in the lower portion of the image. (J, K) Atretic antral follicles (af) served as positive controls for TUNEL; secondary follicle (sf) with no TUNEL staining serves as a negative control. DAPI nuclear counterstain (dim blue). Images in F-I are representative of n = 3-6 follicles per treatment group. Images in F-K are shown at the same magnification; bar in F = 100 µm.

Whole follicle images provide an overview of luteinization and angiogenesis in NTS receptor antagonist–injected follicles (Fig 2). Control follicles (Fig 2A) possessed a thickening granulosa cell layer. Serum progesterone, the classic functional metric of luteinization (1), increased 6-fold in response to hCG administration (Fig 2I). In response to the ovulatory LH surge or hCG, granulosa cells exit cell cycle (26-29). In pre-LH/hCG follicles (Fig 2M) expression of the proliferation antigen Ki67 is prevalent. In contrast, granulosa cells of the control injected follicles showed reduced Ki67 detection (Fig 2N). LH/hCG also induces the expression of prostaglandin synthesis enzymes, most notably PTGS2 (30). In pre-LH/hCG follicles (Fig 2R), granulosa cell expression of PTGS2 was not detected. However, PTGS2 protein was easily detected in granulosa cells of the control injected follicles (Fig 2S) relative to the pre-LH/hCG follicle (Fig 2R). The surrounding stroma contained vessels previously shown to give rise to the developing capillary network of the luteinizing follicle (Fig 2E) (18). New capillary formation was evident when endothelial cells were identified by staining for VWF (Fig 3A). Endothelial cells were visible branching from established stromal vessels (Fig 3A, arrows) as well as between luteinizing granulosa cells from the former basal lamina to very near the antral edge of the luteinizing tissue (Fig 3A, double arrowheads).

Follicles injected with SR142948 were well luteinized, with a thickened layer of granulosa cells (Fig 2B and 2F). Serum progesterone increased 6-fold after hCG administration (Fig 2J), comparable to progesterone levels in control animals (Fig 2I). Granulosa cell expression of Ki67 decreased (Fig 2O) and PTGS2 increased (Fig 2T), similar to control injected follicles (Fig 2N and 2S). Endothelial cells of newly forming capillaries reached near the antral edge of the luteinizing granulosa cell layer, similar to controls (compare control with SR142948 in Fig 3C-3E). A notable difference between control follicles and SR142948-injected follicles was the presence of extravascular red blood cells within (Fig 2F, arrowheads) and at the antral edge (Fig 2F, arrows) of the luteinizing granulosa cell layer.

Follicles injected with SR48692 showed limited luteinization. The granulosa cell layer was thinner when compared with controls (Fig 3C, compare Fig 2E with 2G or Fig 3A with 3B). Serum progesterone increased modestly (1.7-fold) after SR48692 injection and hCG administration, but this increase in serum progesterone was not sustained through day 5 (2 days after hCG; Fig 2K). Ki67 protein expression by granulosa cells decreased relative to pre-LH/hCG follicles (Fig 2P) and PTGS2 expression was increased (Fig 2U), similar to control injected follicles (Fig 2N, 2S). Angiogenesis was very limited, with endothelial cells noted in the stroma surrounding the SR48692-injected follicle (Fig 3B, arrowheads), but no branching evident from stromal vessels (Fig 3B, arrow), and limited endothelial cell progression into the granulosa cell layer (Fig 3D). Extravascular red blood cell accumulation was noted within the luteinizing granulosa cell layer (Fig 2G, arrowhead) and in the antral space of follicles injected with SR48692 (Fig 2G, arrow and Fig 3B, RBCs as indicated).

Follicles injected with AF38469 were modestly luteinized, with a robust 4-fold increase in serum progesterone after hCG administration (Fig 2L), reduction in Ki67 protein (Fig 2Q), and increase in PTGS2 protein (Fig 2V), similar to control injected follicles (Fig 2N and 2S). Overall thickness of the granulosa cell layer was less than in control follicles and not different from follicles injected with either SR142948 or SR48692 (Fig 3C). Vascular endothelial cells were observed within the luteinizing granulosa cell layer, from the basal lamina and approaching the antral edge, comparable to control and SR142948-injected follicles (Fig 3D). Of note, numerous, large accumulations of extravascular red blood cells were present within the luteinizing granulosa cell layer of follicles injected with AF38469 (Fig 2H, arrowheads), and extensive pooling of red blood cells was noted in the antral spaces of AF38469-injected follicles (Fig 2H, arrows).

Limited luteinization and angiogenesis, coupled with low serum progesterone in animals from the SR48692 follicle injection group, raised the question of follicle health after intrafollicular injection. TUNEL was performed to detect DNA fragmentation, characteristic of apoptotic cell death (31). TUNEL staining was not detected in any cell type of control follicles (Fig 3F) or in follicles injected with SR142948 (Fig 3G), SR48692 (Fig 3H), or AF38469 (Fig 3I). Fragmented DNA was detected in atretic antral follicles (Fig 3J and 3K) but not healthy secondary follicles (Fig 3J), which served as positive and negative controls for the TUNEL assay, respectively.

Overall, key effects of NTS receptor blockade on the ovulatory process were noted. Structural luteinization, as measured by the thickness of the granulosa cell layer, was reduced after injection of either the NTSR1 antagonist SR48692 or the SORT1 antagonist AF38469 when compared to control injected follicles (Fig 3C). On any study day, serum progesterone was not different between treatment groups (ANOVA *P* > .05 on each day when all 4 treatment groups were compared) (Fig 2I-2L). However, within each treatment group, only animals with SR48692-injected follicles failed to attain and sustain a robust increase in serum progesterone after hCG administration (Fig 2K). Follicles injected with either vehicle (control), SR142984, or AF38469 showed evidence of new capillary formation in the luteinizing granulosa cell layer (Fig 3E), with endothelial cells observed near the antral edge of the luteinizing granulosa cell layer (Fig 3E). Follicles injected with SR48692 showed limited, if any, evidence of follicular angiogenesis (Fig 3E). Importantly, follicles injected with SR142948, SR48692, or AF38469 contained pools of extravascular red blood cells within the luteinizing granulosa cell layer and in the follicle antrum (Fig 2), neither of which were observed in control follicles.

### NTS-Mediated Permeability Changes are Dependent on Both NTSR1 and SORT1

**Figure 4.**
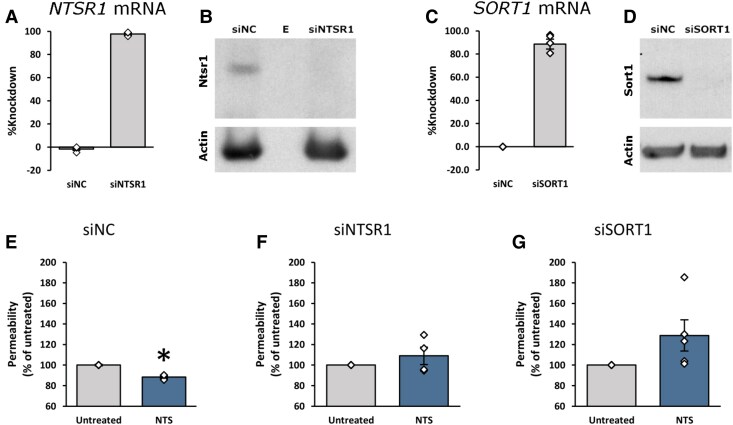
NTSR1 and SORT1 mediate mOMEC monolayer permeability in vitro. (A-D) mOMECs were transfected with either a nontargeting control siRNA (siNC) or siRNAs targeting NTSR1 (siNTSR1) or SORT1 (siSORT1) prior to RNA and protein isolation followed by assessment of mRNA knockdown by qPCR (A, C) or protein knockdown by Western blot (B, D). In B, lane labeled “E” was empty (no protein loaded). For A and C, diamonds represent individual data points. Data are presented as mean ± SEM, n = 4 mOMEC lines/treatment group. (E-G) mOMECs were transfected with either a nontargeting control siRNA (siNC) or siRNAs targeting NTSR1 (siNTSR1) or SORT1 (siSORT1) were treated with vehicle (untreated) or NTS and assessed by trans-well permeability assay. Diamonds represent individual data points. Within each panel, groups with an asterisk are significantly different from untreated control by 2-tailed paired t-test, *P* < .05 (E, *P* = .0098). Data are presented as mean ± SEM, n = 4-5 mOMEC lines/treatment group.

Pooling of red blood cells outside the vasculature of NTS receptor antagonist injected follicles and our prior studies (4, 7) indicate that NTS acts at NTS receptors to regulate vascular permeability. To determine if NTSR1 or SORT1 are necessary for NTS-mediated changes in vascular permeability, primary mOMECs were studied in vitro (18). Expression of key NTS receptors (NTSR1 or SORT1) was reduced using siRNA silencing prior to NTS treatment and assessment of permeability. A nontargeting siRNA (siNC) was used as a control. Knockdown of *NTSR1* mRNA was >95% (Fig 4A), and Western blot confirmed knockdown of NTSR1 protein (Fig 4B). Knockdown of *SORT1* mRNA was >85% (Fig 4C), and Western blot confirmed knockdown of SORT1 protein (Fig 4D). Use of the nontargeting siRNA (siNC) confirmed expression of NTSR1 (Fig 4B) and SORT1 (Fig 4D) proteins in the absence of silencing. In cells transfected with siNC, NTS treatment decreased the permeability of mOMEC monolayers (Fig 4E), consistent with our previous report (7). siRNA knockdown of NTSR1 (Fig 4F) or SORT1 (Fig 4G) eliminated the NTS-regulated decrease in permeability relative to cells which did not receive NTS treatment (untreated). While knockdown of SORT1 resulted in a trend towards an increase in permeability after NTS treatment, this increase was not significant relative to untreated control cell permeability (Fig 4G).

### NTS-Mediated mOMEC Migration is Dependent on Both NTSR1 and SORT1

**Figure 5.**
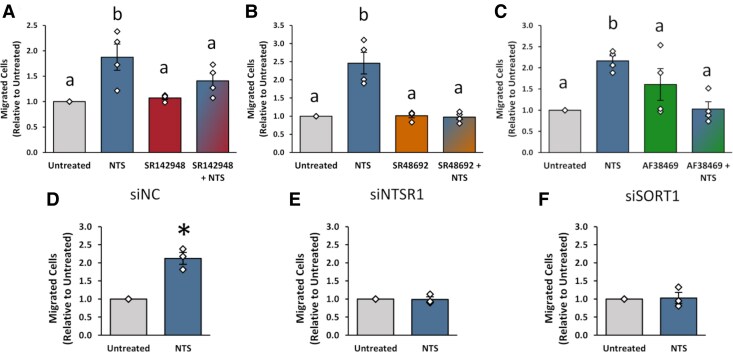
NTSR1 and SORT1 mediate mOMEC migration in vitro. (A-C) mOMECs were untreated or treated with NTS, NTS receptor antagonist alone (SR142948, SR48692, or AF38469), or NTS + NTS receptor antagonist as indicated, then assessed for migration. For each mOMEC line, migration in response to treatments is presented relative to the untreated control, which was set equal to 1.0. Diamonds represent individual data points. Within each panel, groups with no common letter are different by repeated measures ANOVA (A, *P* = .0089; B, *P* = .0001; C, *P* = .0098) and Duncan's post hoc test, *P* < .05. Data are presented as mean ± SEM, n = 4 mOMEC lines/treatment group. (D-F) mOMECs were transfected with either a nontargeting control siRNA (siNC, D) or siRNAs targeting NTSR1 (siNTSR1, E) or SORT1 (siSORT1, F) prior to treatment with either vehicle (untreated) or NTS and assessed for migration. Diamonds represent individual data points. Within each panel, groups with an asterisk are significantly different from untreated control by 2-tailed paired t-test, *P* < .05 (D, *P* = .0400). Data are presented as mean ± SEM, n = 3 mOMEC lines/treatment group.

Our prior studies demonstrated that NTS acts at mOMECs to stimulate migration (4). In the present study, endothelial cell invasion into the luteinizing follicle in vivo was reduced by treatment with either SR48692 or AF38469 (Fig 3D). To identify which receptor(s) mediate NTS-stimulated migration in mOMECs in vitro, cells were either untreated or pretreated with SR142948, SR48692, or AF38469 prior to treatment with NTS. NTS alone increased mOMEC migration relative to untreated cells (Fig 5A-5C), consistent with our previous findings (4). Pretreatment with either SR142948 (Fig 5A), SR48692 (Fig 5B), or AF38469 (Fig 5C) abrogated the NTS effect on migration and resulted in no change relative to untreated cells. While treatment with AF38469 alone resulted in a trend towards increased migration, this increase was not significant relative to untreated cells (Fig 5C).

To further explore the role of individual receptors in mOMEC migration, NTSR1 or SORT1 were knocked down using siRNA silencing prior to NTS treatment and assessment of migration. In cells transfected with siNC, NTS treatment increased migration compared to the untreated control (Fig 5D). However, in NTSR1- and SORT1-silenced mOMECs, NTS was unable to increase migration (Fig 5E and 5F), further supporting involvement of NTSR1 and SORT1 in NTS-stimulated mOMEC migration.

## Discussion

This study examined the ovulatory role of the LH-stimulated paracrine mediator NTS in a primate species, the cynomolgus macaque, which has ovulatory processes that are very similar to those of women. In our prior report, intrafollicular neutralization of NTS reduced follicle rupture, altered luteinization, and resulted in excessive red blood cell accumulation in the follicle antrum (4). The present study was conducted to evaluate the role of individual NTS receptors in mediating these critical actions of NTS in primate ovulation.

Follicle rupture is a complex process that involves tightly orchestrated removal of multiple cell layers and extracellular matrix reorganization in a single, focal area of the follicle wall (1). NTS action to facilitate follicle rupture was mediated by NTSR1 and SORT1. For these studies, rupture was defined as a breach in the ovarian surface that connected the follicle antrum to the exterior of the ovary as such a breach is needed for transit of a cumulus oocyte complex into the oviduct. The general NTS receptor antagonist SR142948 or the SORT1 antagonist AF38469 resulted in small or absent rupture sites 48 hour after hCG. Follicles injected with the NTSR1 selective antagonist, SR48692, did not experience true follicle rupture by 48 hours after hCG. Interestingly, all follicles injected with SR48692 showed histological evidence of a breach in the granulosa cell layer connecting the follicle antrum to the surrounding ovarian stroma, which was termed as an aberrant rupture. One SR48692-injected follicle had multiple aberrant ruptures. These findings support identification of NTSR1 as the key receptor to mediate NTS regulation of rupture site location at the follicle apex. Similar dysfunctional rupture at 48 hours after hCG (including absent, aberrant, and multiple rupture sites) has been previously reported after blockade of intrafollicular prostaglandin synthesis or in the presence of prostaglandin receptor antagonists (17, 32, 33). Follicular prostaglandins were not measured in the present study, but NTS receptor blockade did not alter the prostaglandin synthesis/response pathway in cultured mouse granulosa cells (6). Apical vasoconstriction has been associated with follicle rupture (34), and possible disruption of apical vasoconstriction was noted in follicles injected with the SORT1 antagonist, AF38469. Additional factors, including changes in immune cell functions (35), have also been associated with follicle rupture. While a complete picture of how the ovulatory follicle ruptures at a single site remains elusive, this study demonstrates a role for NTS, acting via NTSR1, in this complex process.

NTS acts via its receptors to promote luteinization, a multifaceted process by which the ovulatory follicle transforms into the corpus luteum (1). Functional luteinization is characterized primarily by a consistent increase in progesterone synthesis after the LH surge or administration of an ovulatory hCG stimulus (2). Follicles injected with vehicle (control), SR142948, or AF38469 showed the typical, sustained rise in serum progesterone after systemic hCG administration. In contrast, follicles injected with the NTSR1 antagonist SR48692 showed only a modest increase in progesterone after hCG administration, and this increase in progesterone was not sustained at the time of ovariectomy. LH/hCG also results in elevated follicular PGE2 levels, due to enhanced expression of prostaglandin synthesis enzymes in luteinizing granulosa cells, most notably PTGS2 (30). In our studies, hCG increased PTGS2 expression in granulosa cells of all treatment groups. A consistent relationship between follicular NTS and progesterone has not been noted previously in vivo (4, 14) or in vitro (6). A strength of the present report is that structural luteinization was assessed quantitatively by expansion of the granulosa cells into the antrum concomitantly with the invasion of capillaries from the stroma into the granulosa cell layer. In response to LH/hCG, granulosa cells exit cell cycle and exhibit notable hypertrophy due to lipid accumulation which results in a thickening of the granulosa cell layer (1). Granulosa cells from all injection groups showed decreased Ki67 expression, indicating cell cycle exit. Follicle injection with either SR48692 or AF38469 decreased granulosa cell layer thickness and endothelial cell migration into the granulosa cell layer. While both NTSR1 and SORT1 are implicated in structural luteinization, the effects of the NTSR1 antagonist SR48692 were most pronounced. Since both structural and functional luteinization were compromised with the NTSR1 antagonist SR48692, the concern was raised that follicle health was negatively impacted. However, TUNEL staining was not observed in control follicles nor any antagonist-injected follicles, demonstrating that the observed changes in luteinization were not the result of apoptotic cellular changes.

Vascular permeability in the ovulatory follicle increases after the LH surge (36, 37), and the present study implicates LH-stimulated NTS as a paracrine mediator of follicular vascular permeability. Blockade of NTS action at NTSR1, SORT1, or both NTS receptors resulted in excessive extravascular red blood cell accumulation in the granulosa cell layer and in the follicle antrum in vivo. Studies in the present report also demonstrate that both NTSR1 and SORT1 are required to mediate NTS's role in maintaining low permeability in mOMEC monolayers in vitro. There is marked heterogeneity between vascular endothelial cells from various vascular beds of the body (38), which is further demonstrated by their varied responses to NTS signaling. In contrast to the current study, NTS has been shown to increase permeability in many vascular endothelial cell populations (39-42). However, NTS does not affect permeability in vascular endothelial cells of the blood–brain barrier (40). Rapid angiogenesis during ovulation and luteinization is typical of the ovulatory follicle but would be highly unusual in other organs (18). New capillary formation requires adjacent endothelial cells of stable vessels to loosen their cell–cell contacts, allowing endothelial cells to migrate away from the parent vessel to form a new capillary (43). Connections between adjacent endothelial cells can be modulated by several types of cell–cell junctions (44), and we recently demonstrated that NTS decreased ovarian endothelial cell permeability by modulation of CDH2 and CDH6 adherens junctions (7). In addition, NTS may work in concert with other vascular growth regulators to optimize vascular permeability (45) and permit new capillary formation while preventing loss of vessel integrity during ovulatory angiogenesis.

NTS promotes endothelial cell migration via NTSR1 and SORT1. Antagonists against NTSR1 or SORT1 reduced migration of endothelial cells into the luteinizing granulosa cell layer in vivo. Use of siRNA knockdowns and receptor selective antagonists in this study confirmed that NTSR1 and SORT1 can each promote migration of mOMECs in vitro. NTS has been reported to stimulate migration of cells in numerous cancer types (46-48), microglia (49), macrophages (50), and vascular endothelial cells (51-53). In other vascular endothelial cells, NTS stimulates angiogenic migration through modulation of the expression of proangiogenic genes (51-53). However, in a previous study by our group, no significant changes in mOMEC mRNA expression were seen after treatment with NTS (7), indicating NTS-mediated actions are likely controlled by nontranscriptional signaling cascades. Overall, our findings indicate that NTSR1 and SORT1 play a crucial role in mediating angiogenesis during ovulation.

This study utilized NTS receptor antagonists to selectively block signaling through their respective receptors. To minimize off-target effects, antagonist doses were carefully selected based on prior experimental reports of SR142948 (54-57), SR48692 (58-62), and AF38469 (61, 63, 64). Complementary strategies were also employed to modulate NTS receptor activity in both in vivo and in vitro settings. In vivo, our group previously utilized an NTS-neutralizing antibody and observed a comparable phenotype to the NTS receptor antagonist injected follicles in the present study (4, 7). In vitro, the effects of the NTS receptor antagonists aligned with siRNA knockdown of either NTSR1 or SORT1. Collectively, these findings suggest that the observed changes in response to the NTS receptor antagonists in this study are specifically due to reduced NTS signaling through their respective receptors.

Our data do raise the question of why the effects of the general NTS receptor antagonist SR142948 appear modest in contrast with the impact of the NTSR1 antagonist SR48692 or the SORT1 antagonist AF38469 in vivo. The complexity of the monkey follicle injection model does not permit optimization of antagonist concentrations in vivo. It is possible that a modestly higher concentration of SR142948 would more completely block NTS action and have a greater impact on ovulation. While the effects of follicle injection with SR142948 were less pronounced, SR142948 did cause ovulatory changes, including reduced follicle rupture rate, reduced follicle rupture size, and extravascular pooling of red blood cells. Finally, in vitro studies showing similar effects of SR142948, SR48692, AF38469, NTSR1 knockdown, and SORT1 knockdown on mOMEC migration support the concept that both NTSR1 and SORT1 mediate key actions of NTS to induce ovulatory changes.

The studies presented here implicate NTS as an important mediator of ovulation via follicle rupture, luteinization and ovulatory angiogenesis. Analysis of the structure/function of injected follicles and in vitro studies of mOMECs suggests key roles for NTSR1 and SORT1 to mediate NTS action in these ovulatory events. Very small or absent rupture sites were seen with the NTSR1 antagonist or NTS-neutralizing antibody (4), suggesting that rupture may be most dependent on NTSR1. The NTSR1 antagonist also negatively impacted both structural and functional luteinization. Our in vitro studies demonstrate that mOMECs utilize both NTSR1 and SORT1 to mediate NTS-regulated changes in permeability and migration, key events of ovulatory angiogenesis. While not quantifiable, very bloody rupture sites and extensive red blood cell accumulation within the follicle antrum were noted with the SORT1 antagonist or NTS-neutralizing antibody (4), supporting the concept that SORT1 may be a critical receptor for NTS-stimulated vascular changes during ovulation. NTS-stimulated signal transduction via NTSR1 and SORT1 is poorly understood and likely cell and tissue specific (8). In addition, reports of SORT1 acting in heterodimeric receptor complexes with NTSR1 (65) provide additional potential signaling mechanisms for NTS in the cells of the ovulatory follicle. While the present report focused on direct action of NTS at vascular endothelial cells, NTS is also likely to act at granulosa, theca, and other follicular cell types to influence ovulatory changes in vivo and in vitro. Overall, this study identifies NTSR1 and SORT1 as potential targets to promote fertility and/or the development of novel contraceptive agents.

## Data Availability

Original data generated and analyzed during this study are included in this published article or in the data repositories listed in References.

## Abbreviations

ANOVA: analysis of variance
FSH: follicle-stimulating hormone
GnRH: gonadotropin releasing hormone
hCG: human chorionic gonadotropin
LH: luteinizing hormone
mOMEC: monkey ovarian microvascular endothelial cell
NTS: neurotensin
NTSR1: neurotensin receptor 1
qPCR: quantitative polymerase chain reaction
SHRP: horseradish peroxidase conjugated streptavidin
TUNEL: Terminal deoxynucleotidyl transferase dUTP nick-end labelling
VWF: Von Willebrand factor
